# Aluminum Chloride‐Graphite Batteries with Flexible Current Collectors Prepared from Earth‐Abundant Elements

**DOI:** 10.1002/advs.201700712

**Published:** 2018-01-22

**Authors:** Shutao Wang, Kostiantyn V. Kravchyk, Alejandro N. Filippin, Ulrich Müller, Ayodhya N. Tiwari, Stephan Buecheler, Maryna I. Bodnarchuk, Maksym V. Kovalenko

**Affiliations:** ^1^ Laboratory of Inorganic Chemistry Department of Chemistry and Applied Biosciences ETH Zürich Vladimir‐Prelog‐Weg 1 CH‐8093 Zürich Switzerland; ^2^ Laboratory for Thin Films and Photovoltaics Empa – Swiss Federal Laboratories for Materials Science and Technology Überlandstrasse 129 CH‐8600 Dübendorf Switzerland; ^3^ Laboratory for Nanoscale Materials Science Empa – Swiss Federal Laboratories for Materials Science and Technology Überlandstrasse 129 CH‐8600 Dübendorf Switzerland

**Keywords:** aluminum chloride graphite batteries, current collectors, energy density, graphite, titanium nitride

## Abstract

In the search for low‐cost and large‐scale stationary storage of electricity, nonaqueous aluminum chloride‐graphite batteries (AlCl_3_‐GBs) have received much attention due to the high natural abundances of their primary constituents, facile manufacturing, and high energy densities. Much research has focused on the judicious selection of graphite cathode materials, leading to the most notable recent advances in the performance of AlCl_3_‐GBs. However, the major obstacle to commercializing this technology is the lack of oxidatively stable, inexpensive current collectors that can operate in chloroaluminate ionic liquids and are composed of earth‐abundant elements. This study presents the use of titanium nitride (TiN) as a compelling material for this purpose. Flexible current collectors can be fabricated by coating TiN on stainless steel or flexible polyimide substrates by low‐cost, rapid, scalable methods such as magnetron sputtering. When these current collectors are used in AlCl_3_‐GB coin or pouch cells, stable cathodic operation is observed at voltages of up to 2.5 V versus Al^3+^/Al. Furthermore, these batteries have a high coulombic efficiency of 99.5%, power density of 4500 W kg^−1^, and cyclability of at least 500 cycles.

Interest in grid‐scale batteries for stationary electricity storage has soared over the past decade due to the growing contributions of variable energy sources, such as wind and solar, to total global electricity production.^1^, ^2^ In highly simplified terms, the economic feasibility of grid‐scale electricity storage can be determined by comparing the capital costs of stored energy per cycle (¢ kW^−1^ h^−1^ cycle^−1^) of new technologies to those of traditional pumped hydroelectric storage (0.1–1.4 ¢ kW^−1^ h^−1^ cycle^−1^), which is currently the predominant technology.^3^ The energy densities of grid‐scale battery systems, typically expressed in Wh kg^−1^, are less important than those of rechargeable batteries in portable electronics and mobile applications. The strict cost requirements can only be met when a battery is exclusively composed of inexpensive, earth‐abundant, easy‐to‐produce components. For example, the use of Li ions can be questionable due to the limited, nonuniform natural abundance of this element in the Earth's crust.^4^, ^5^ Hence, research has increasingly focused on the electrochemistries of Na,^6^, ^7^, ^8^, ^9^, ^10^, ^11^, ^12^, ^13^, ^14^, ^15^, ^16^, ^17^, ^18^, ^19^ K,^20^, ^21^, ^22^, ^23^, ^24^ Ca,^25^, ^26^, ^27^, ^28^ Mg,^29^, ^30^, ^31^, ^32^ and Al.^33^, ^34^, ^35^, ^36^, ^37^, ^38^, ^39^ In particular, aluminum chloride‐graphite batteries (AlCl_3_‐GBs) have attracted considerable attention because they are composed of highly abundant elements (H, O, N, C, and Al) and have appropriate energy densities (30–70 Wh kg^−1^).^33^, ^37^, ^40^, ^41^ The basic architecture of an AlCl_3_‐GB consists of a metallic aluminum current collector, AlCl_3_‐[EMIM]Cl (1‐ethyl‐3‐methylimidazolium chloride) ionic liquid anolyte and graphite cathode, as shown in **Figure** **1**a. To date, natural graphite flakes,^40^, ^41^ kish graphite flakes,^43^ graphitic foams,^33^, ^44^ graphene nanoribbons,^45^ few‐layer graphene aerogels,^46^ graphene mesh network,^47^ large‐sized few‐layer graphene,^48^ and carbon paper (of graphitic nature),^49^, ^50^, ^51^ have been employed as the cathode material, delivering capacities of 60–150 mA h g^−1^ and average discharge voltages of 1.7–2 V.

**Figure 1:**
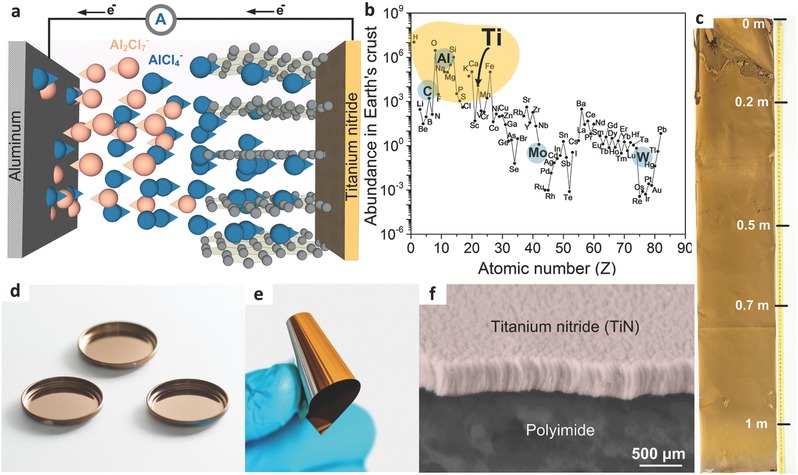
Aluminum chloride‐graphite battery with a TiN current collector. a) Schematic of the charging process. b) Element abundances in the Earth's crust (normalized per 10^6^ atoms of Si) (the yellow region indicates rock‐forming elements) (adapted from ref. 42). Photographs of the c,e) TiN‐coated (500 nm film thickness) polyimide and d) stainless steel substrates. f) Cross‐sectional SEM image of TiN deposited on the polyimide substrate.

In contrast, the focus of this work is to find inexpensive stable materials that are suitable cathode current collectors, addressing the most pressing limitation of AlCl_3_‐GBs. Common metals, such as Al and stainless steel (SS), are rapidly corroded in AlCl_3_‐based ionic liquids at the high voltages employed during cathode operation. It was determined that the corrosion onset potential of 2.5 V versus Al^3+^/Al in AlCl_3_‐[EMIM]Cl is equivalent to a potential of 4.8 V versus Li^+^/Li (see Figure S1a–d, Supporting Information). Even the noble metal gold is rapidly etched at 2 V versus Al^3+^/Al (Figure S2, Supporting Information). Hence, the only stable current collectors employed to date were based on tungsten (W), molybdenum (Mo), and glassy carbon (GC).

Herein, we report that titanium nitride (TiN), a strongly electrically conductive material composed of abundant elements, is ideally suited for use as an AlCl_3_‐GB current collector due to its high oxidative stability in the ionic liquid AlCl_3_‐[EMIM]Cl at potentials of up to at least 2.5 V versus Al^3+^/Al. TiN films can be deposited on stainless steel and flexible polyimide substrates for use in coin and pouch cell batteries, respectively. The superior stability of these materials toward electrochemical oxidation enables a higher coulombic efficiency of ≈99.5% to be achieved at currents of 1–10 A g^−1^. The AlCl_3_‐GBs with TiN current collectors also exhibit a high power density of 4500 W kg^−1^ at a high graphite loading (10 mg cm^−2^) and are stable for at least 500 cycles.

The natural abundance of Ti in the Earth's crust is 4–5 orders of magnitude higher than those of Mo and W (Figure 1b), and the nitrogen supply is essentially unlimited. TiN has been used as a “diffusion barrier metal” in microelectronics and as a protective and/or hard coating due to its high corrosion resistance and good edge retention.^52^ In this work, TiN current collector films were magnetron sputtered onto stainless steel and flexible polyimide substrates under a nitrogen–argon atmosphere using a Ti target (Figure 1c,d). Magnetron sputtering enables the scalable and inexpensive fabrication of TiN films on a square meter scale (Figure 1c). X‐ray diffraction (XRD) analysis (Figure S3, Supporting Information) indicates the formation of a pure‐phase TiN film on the polyimide substrate (space group $Fm\bar{3}m$, *a* = 0.4241 nm, JCPDS 038‐1420).^53^ The XRD pattern of the TiN films shows that they are highly oriented in the (111) direction, and the cross‐sectional scanning electron microscopy (SEM) images shown in Figure 1f and Figure S4 (Supporting Information) reveal their columnar morphology. Top‐view SEM images of the samples show that the substrates are completely covered by the nonporous, pin‐hole‐free, and continuous films (Figure S5, Supporting Information). The use of the polyimide substrate imparts good flexibility to the current collector (Figure 1e). Importantly, repeatedly bending the 20–1000 nm thick TiN films does not result in a decrease in the electrical conductivity, as illustrated in Video S1 (Supporting Information).

The electrochemical stability of the TiN current collector in a chloroaluminate ionic liquid was evaluated using the most acidic ionic liquid formulation, that is, an AlCl_3_:[EMIM]Cl ratio (denoted *r*) of 2.0. It should be noted that this *r* value, which is the highest value at which the mixture is still completely liquid, yields a theoretical energy density of 65–70 Wh kg^−1^.^41^, ^43^ At lower *r* values of 1.1 and 1.3, the theoretical energy densities are only 13 and 33 Wh kg^−1^, respectively. The charge storage capacity of the graphite electrode has a relatively minor effect on the overall theoretical energy density of the battery, as demonstrated and discussed in detail in our earlier publications.^41^, ^43^ **Figure** **2**a shows the cyclic voltammetry (CV) curves for various current collectors in the chloroaluminate ionic liquid, and the results are summarized in Figure 2b. The onset of electrochemical oxidation occurs at 0.62, 0.92, 1, and 1.1 V versus Al^3+^/Al for chromium (Cr), SS, Al, and Ti metals, respectively. Furthermore, even gold and platinum are not stable at potentials above 2 V. The oxidative stability of TiN in AlCl_3_‐[EMIM]Cl exceeds those of Mo, W, and GC. The inset in Figure 2a shows the measured current for the TiN current collector on a logarithmic scale as a function of the applied voltage. The linear part of the TiN current–potential relationship can be well fitted by the Tafel equation, which confirms the absence of any parasitic oxidation reactions at potentials of up to 2.5 V

(1) $$i=i_{0}\;\exp\frac{n\left(E-\varepsilon \right)}{RT}$$

where *i* and *i*_0_ are the measured current and exchange current density, respectively; *n* is the number of electrons transferred in the elemental redox reaction; *E* and ε are the applied and standard redox potentials, respectively; *R* is the gas constant; and *T* is the temperature. A chromium nitride (Cr_2_N) current collector also exhibits a similar oxidative stability (Figure 2a). The increase in the current above 2.5–3 V versus Al^3+^/Al for the TiN and Cr_2_N current collectors is primarily due to the oxidation of the ionic liquid, which results in Cl_2_ gas evolution.^54^

**Figure 2:**
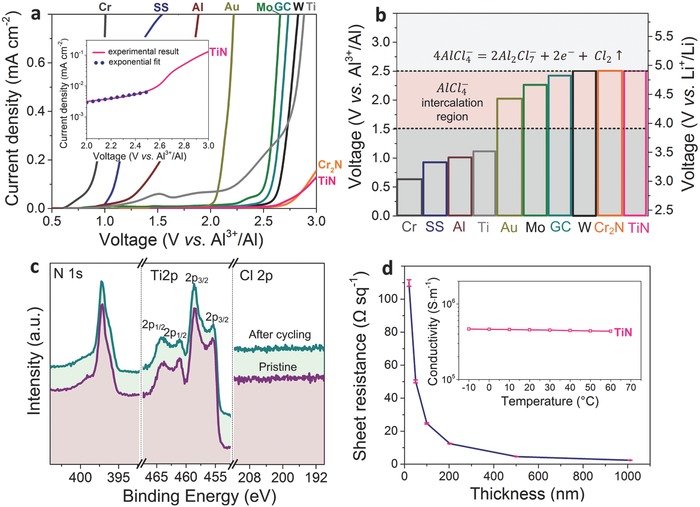
a) Cyclic voltammetry curves for various current collectors measured in AlCl_3_‐[EMIM]Cl (*r* = 2) at a rate of 10 mV s^−1^ (inset: current–potential relationship of the TiN current collector on a logarithmic scale). b) Illustration of the oxidative stabilities of various current collector materials in AlCl_3_‐[EMIM]Cl (*r* = 2) in terms of the voltage versus Al^3+^/Al and Li^+^/Li. c) XPS data before and after cyclic voltammetry measurements of the TiN current collector in AlCl_3_‐[EMIM]Cl (*r* = 2, 1000 cycles within the range of 1–2.5 V vs Al^3+^/Al). d) Dependence of the TiN sheet resistance, which was measured by a four‐point probe technique at 25 °C, on the film thickness (inset: temperature dependence of the TiN electrical conductivity).

To analyze the results for the Al‐based batteries from a broader electrochemical perspective, the exact location of the Al^3+^/Al potential in AlCl_3_‐[EMIM]Cl on the absolute scale versus the standard hydrogen electrode (SHE) was estimated, thus allowing a comparison of the Li and Na electrochemistries. Using specially constructed cells consisting of a β‐alumina solid electrolyte and Na reference electrode (see Figure S1a–d, Supporting Information), the Al^3+^/Al redox reaction (electroplating/electrostripping) is determined to occur at 2 V versus Na^+^/Na (i.e., −0.7 V vs SHE and 2.3 V vs Li^+^/Li), whereas the ionic liquid oxidation process starts at a potential that is ≈2.5 V higher (4.5 V vs Na^+^/Na, 1.8 V vs SHE, and 4.8 V vs Li^+^/Li). These results highlight the high oxidative stability of TiN, and this analysis is also valid at a very slow CV sweep rate of 1 mV s^−1^ (Figure S6, Supporting Information). Consequently, the TiN current collector was also tested in Li and Na electrolytes and was found to outperform common Al and SS current collectors, particularly in Li(Na) bis(fluorosulfonyl)imide (FSI)‐based electrolytes (Figure S7, Supporting Information). Future studies will provide more details about the effectiveness of TiN and similar metal nitrides as alternative current collectors for high‐voltage cathode materials in Li‐ and Na‐ion batteries.

Previously, the high corrosion resistance of TiN was explained by the presence of titanium deficiencies and thus terminal N groups in the surface layer.^55^, ^56^ Nitrogen atoms shield the underlying Ti^3+^ ions, thereby protecting them from oxidation to Ti^4+^. Therefore, TiN has a much higher oxidative stability than pure titanium metal.

The surface of TiN was examined by X‐ray photoelectron spectroscopy (XPS) before and after cyclic voltammetry measurements (1000 cycles within 1–2.5 V vs Al^3+^/Al voltage range) in order to shed the light onto the chemical processes at the surface (Figure 2c). No substantial reactivity can be deduced based on the practically identical Ti 2p and N 1s bands after cycling. Furthermore, no Cl 2p signal can be observed for the cycled TiN collector. The N/Ti ratios on the TiN surface were nearly identical before and after cycling (1.0 ± 0.1 and 1.0 ± 0.1, respectively).

The electrical conductivity was evaluated by measuring the sheet resistances of TiN current collectors with different thicknesses (20–1000 nm) by a four‐point probe method (Figure 2d). The sheet resistance increases dramatically at thicknesses of less than 100 nm, whereas 0.2–1 µm thick films exhibit similar conductivities. The calculated electrical conductivities of all the tested films are in the range of 4–4.5 × 10^5^ S m^−1^. The temperature dependence of the TiN resistivity in the range of −10 to 60 °C was also investigated and found to be linear (inset in Figure 2d), which is consistent with the work of Solovan et al.^57^ The temperature coefficient of electrical resistance (TCR) at 20 °C is as low as 9.7 × 10^−4^ °C^−1^. Assuming that the bulk electrical conductivity in the *ab*‐direction is that of graphite (3 × 10^5^ S m^−1^),^58^ the carrier transport rate is not limited by a 1 µm thick TiN film combined with graphite electrodes with thicknesses of up to 440 µm. In practice, the conductivity of graphite flakes is up to a factor of 3 lower than the idealized bulk value,^59^ further highlighting the potential of TiN for use as a current collector. These considerations are further supported by the observation of the high power density (rate capability), as discussed below.

Full‐cell tests were conducted using coin and pouch cell configurations (**Figure** **3**, and Figure S8, Supporting Information). The AlCl_3_:[EMIM]Cl ratio was 2, and the cathode in the AlCl_3_‐graphite cells consisted of kish graphite flakes in all the experiments.^43^ The electrodes in the coin cells consisted of pressed graphite powder (10 mg cm^−2^), and no binders or conductive additives were used to exclude their effects on the observed characteristics. Figure 3a shows the rate capability and coulombic efficiency data for AlCl_3_‐GBs with TiN and W current collectors. Following our previous work,^43^ a CCCV charging protocol with constant voltage steps at 1.92 and 2.07 V was employed for all the electrochemical measurements, which improved the graphite capacity by 10–20 mA h g^−1^. At high current densities (≥1 A g^−1^), the coulombic efficiencies of the TiN and W current collectors are nearly identical (99.5 and 99.1%, respectively).

**Figure 3:**
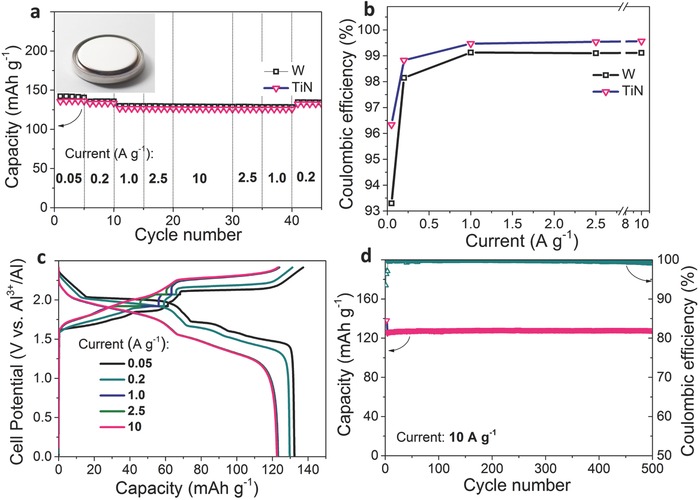
Electrochemical performance of kish graphite flakes in a coin cell with a TiN current collector. The inner surface of the casing at the cathode was coated with TiN (see Figure 1d). a) Rate capabilities, b) coulombic efficiencies, and c) galvanostatic charge–discharge voltage curves measured by a CCCV protocol at various current densities (0.05–10 A g^−1^) in AlCl_3_‐[EMIM]Cl (*r* = 2) (inset in (a): photograph of a coin cell with a TiN current collector). d) Cyclability of kish graphite flakes measured by a CCCV protocol at 10 A g^−1^ (first three cycles measured at 0.05 A g^–1^).

However, the measurements conducted at a slow rate (0.05 A g^−1^) indicate that TiN current collector afford for higher value of 96.3% (Figure 3b). Importantly, graphite flakes measured on both TiN and W current collectors have shown similar charge‐storage capacities of ≈125 mA h g^−1^ at broadly varied current densities (1–10 A g^−1^), in all cases retaining the superb flatness of the voltage profiles (Figure 3c). At the highest current density of 10 A g^−1^, the power density is estimated to be 4500 W kg^−1^. Additionally, the cycling tests (Figure 3d) at this current density show that not only does the capacity remain above 120 mA h g^−1^, but the coulombic efficiency is also 99.5% for at least 500 cycles. Similar electrochemical performance of kish graphite flakes was observed with pouch cell configuration using TiN/polyimide current collector (Figure S8, Supporting Information). It should be noted that stable cycling behavior was also measured for an AlCl_3_‐GB with a Cr_2_N current collector, which has a capacity of 130 mA h g^−1^ at a current density of 500 mA g^−1^ after 100 cycles (Figure S9, Supporting Information). Importantly, the AlCl_3_‐GB does not undergo an irreversible capacity loss in the first cycle, which is a problem inherent to all Li‐ion batteries and is caused by solid‐electrolyte interface formation.

In summary, TiN is an inexpensive current collector material that is easily fabricated from highly abundant elements and can be used in AlCl_3_‐GBs and other types of rechargeable batteries (Li‐ion, Na‐ion, Mg‐ion, etc.). Although nearly all metals, including Au and Pt, are easily corroded by AlCl_3_‐based ionic liquids, TiN current collectors exhibit satisfactory corrosion resistance; in fact, their corrosion resistance is even higher than those of known alternatives (tungsten, molybdenum, and glassy carbon). It was shown that a TiN current collector can be deposited on stainless steel and polyimide substrates for use in coin and pouch cells. The fabricated AlCl_3_‐GB with the TiN current collector exhibits a high power density of at least 4500 W kg^−1^ and cyclability of at least 500 cycles. This work demonstrates the feasibility of commercializing AlCl_3_‐GBs for use as an inexpensive, grid‐level energy storage technology.

## Experimental Section

*Chemicals and Battery Components*: [EMIM]Cl (99%, IoLiTec), AlCl_3_ (99%, granules, Acros), Al foil (MTI Corporation), stainless steel (316S, Hohsen), a polyimide (Kapton), soda lime glass, W plates (MTI Corporation), GC plates (Goodfellow), Cr targets (99.95%, Umicore), Ti targets (99.95%, Umicore), a glass microfiber separator (GF/D, Cat. No. 1823‐257, Whatman), a polyvinylidene fluoride binder (PVDF, Aldrich), and *N*‐methyl‐2‐pyrrolidone (NMP, Fischer) were used as received.

*TiN and Cr_2_N Current Collector Fabrication*: TiN and Cr_2_N were deposited on stainless steel (316L, Hohsen Corp.), polyimide, or soda lime glass substrates by pulsed DC magnetron sputtering using Ti and Cr targets, respectively, under an Ar/N_2_ atmosphere (for TiN: Ar:N_2_ ratio of 82.5:23 (sccm), pressure of 0.5 Pa; for Cr_2_N: Ar:N_2_ ratio of 12.5:7.5 (sccm), pressure of 0.3 Pa). Before each deposition, both the substrate and target were pre‐sputtered for 5 (TiN) or 10 (Cr_2_N) min in pure Ar, and the targets for the TiN and Cr_2_N samples were subsequently poisoned under an 82.5:30 (sccm) or 3:18 (sccm) Ar:N_2_ flow, respectively, for 5 min. The target power was set to 0.58 W cm^−2^ for TiN and 0.32 W cm^−2^ for Cr_2_N, and the temperature was 200 and 300 °C for TiN and Cr_2_N, respectively.

*Four‐Point Probe Resistivity Experiments*: Four‐point probe resistivity measurements of the coated soda lime glass substrates were performed using a NAGY SD‐600 instrument. The error in the measured sheet resistance was less than 2%.

*Temperature Dependence of the TiN Resistivity*: To determine the TiN resistivity at different temperatures, gold contacts (200 nm thick) were deposited by thermal evaporation on the TiN (500 nm thick)/soda lime glass current collectors with the spacing shown in Figure S10 (Supporting Information). The gold (UBS) was evaporated at a pressure of 2 × 10^−4^ Pa and rate of 1.3 Å s^−1^. The gold contact area was etched with a diamond tip to minimize edge effects. The samples were placed in a temperature‐controlled environment, and the resistance between each pair of contacts was determined from the *I*–*V* curves obtained by four‐point probe measurements at a low pressure (800 Pa) in the temperature range −10 to 60 °C using a Keithley 2400 source meter. The sample resistance was obtained using the transmission line method; the data were fitted by linear regression at each temperature, as shown in Figure S11 (Supporting Information) (the slope is the sample resistance). The resistivity was then calculated by multiplying the resistance by the contact length (8 mm) and layer thickness (500 nm).

*Characterization*: SEM images were acquired using a Hitachi S‐4800 FEG‐SEM at a working voltage of 5 kV to minimize electrical charging of the layers. X‐ray diffractograms were obtained using a PANalytical X'Pert PRO diffractometer with Cu Kα radiation in a Bragg–Brentano configuration. XPS measurements were performed using a monochromatic Al Kα X‐ray source (PHI Quantum 2000) at room temperature. The surveys and single scans were performed using pass energies of 117.4 and 29.35 eV, respectively. The atomic concentrations were determined using the MultiPak software. Cyclic voltammetry measurements of the TiN‐coated polyimide, aluminum, and stainless steel current collectors were performed on an MPG2 multichannel workstation (Bio‐Logic).

*Chloroaluminate Ionic Liquid Preparation*: The [EMIM]Cl‐based ionic liquid was prepared by slowly mixing the solid [EMIM]Cl powder and AlCl_3_ granules in an argon‐filled glove box. During mixing, an isothermal reaction occurs to give a light‐yellow liquid, which was subsequently treated with Al foil at 150 °C for 6 h until it was nearly colorless.

*Graphite Cathode Preparation*: Large commercial graphite flakes (0.2 g, grade 200, Graphene Supermarket) were placed in a 4 mL glass vial with 3.5 mL of ethanol and sonicated for 30 min (10% power) using a Sonopuls HD2200 ultrasonic homogenizer. Then, the sonicated kish graphite flakes were washed three times with ethanol and dried under vacuum at 80 °C for 12 h.

*Assembly and Testing of the AlCl_3_‐Graphite Batteries*: No binders or solvents were used to prepare the electrodes used in the coin and pouch‐type cell batteries. The graphitic material (10 mg over ≈1 cm^2^) was homogeneously distributed and pressed on the surface of TiN (Cr_2_N)‐coated stainless steel cap or TiN/polyimide current collector. Both the coin and pouch cells were assembled in an argon‐filled glove box (O_2_ < 1 ppm, H_2_O < 1 ppm) using a glass fiber separator soaked with AlCl_3_‐[EMIM]Cl. Aluminum foil was used as both the reference and counter electrodes. These cells were cycled between 0.01 and 2.415 V on an MPG2 multichannel workstation (Bio‐Logic). A CCCV protocol was used at voltages of 1.92 and 2.07 V until the current decreased to 10% of the initial value.

## Conflict of Interest

The authors declare no conflict of interest.

## Supporting information

Supplementary

Supplementary
