# Supplementary material for: Aluminum Chloride‐Graphite Batteries with Flexible Current Collectors Prepared from Earth‐Abundant Elements
Source: Adv Sci (Weinh). 2018 Jan 22;5(4):1700712. doi: 10.1002/advs.201700712 (PMC5908378; doi:10.1002/advs.201700712)
Supplement: Supplementary file 1 — Supplementary [file ADVS-5-1700712-s002.pdf]

Copyright WILEY-VCH Verlag GmbH & Co. KGaA, 69469 Weinheim, Germany, 2018.

ADVANCED SCIENCE
Open Access

Supporting Information

for *Adv. Sci.,* DOI: 10.1002/advs.201700712

Aluminum Chloride-Graphite Batteries with Flexible Current Collectors Prepared from Earth-Abundant Elements

*Shutao Wang, Kostiantyn V. Kravchyk, Alejandro N. Filippin, Ulrich Müller, Ayodhya N. Tiwari, Stephan Buecheler, Maryna I. Bodnarchuk, and Maksym V. Kovalenko**

WILEY-VCH

Copyright WILEY-VCH Verlag GmbH & Co. KGaA, 69469 Weinheim, Germany, 2017.

Supporting Information

Aluminum Chloride-graphite Batteries with Flexible Current Collectors Prepared from Earth-abundant Elements

Shutao Wang,[†,‡,#] *Kostiantyn V. Kravchyk,*[†,‡,#] *Alejandro N. Filippin,*[‡,#] *Ulrich Müller,*[£] *Ayodhya N. Tiwari,*[‡] *Stephan Buecheler,*[‡] *Maryna I. Bodnarchuk,*[‡] *and Maksym V. Kovalenko*[*,†,‡]

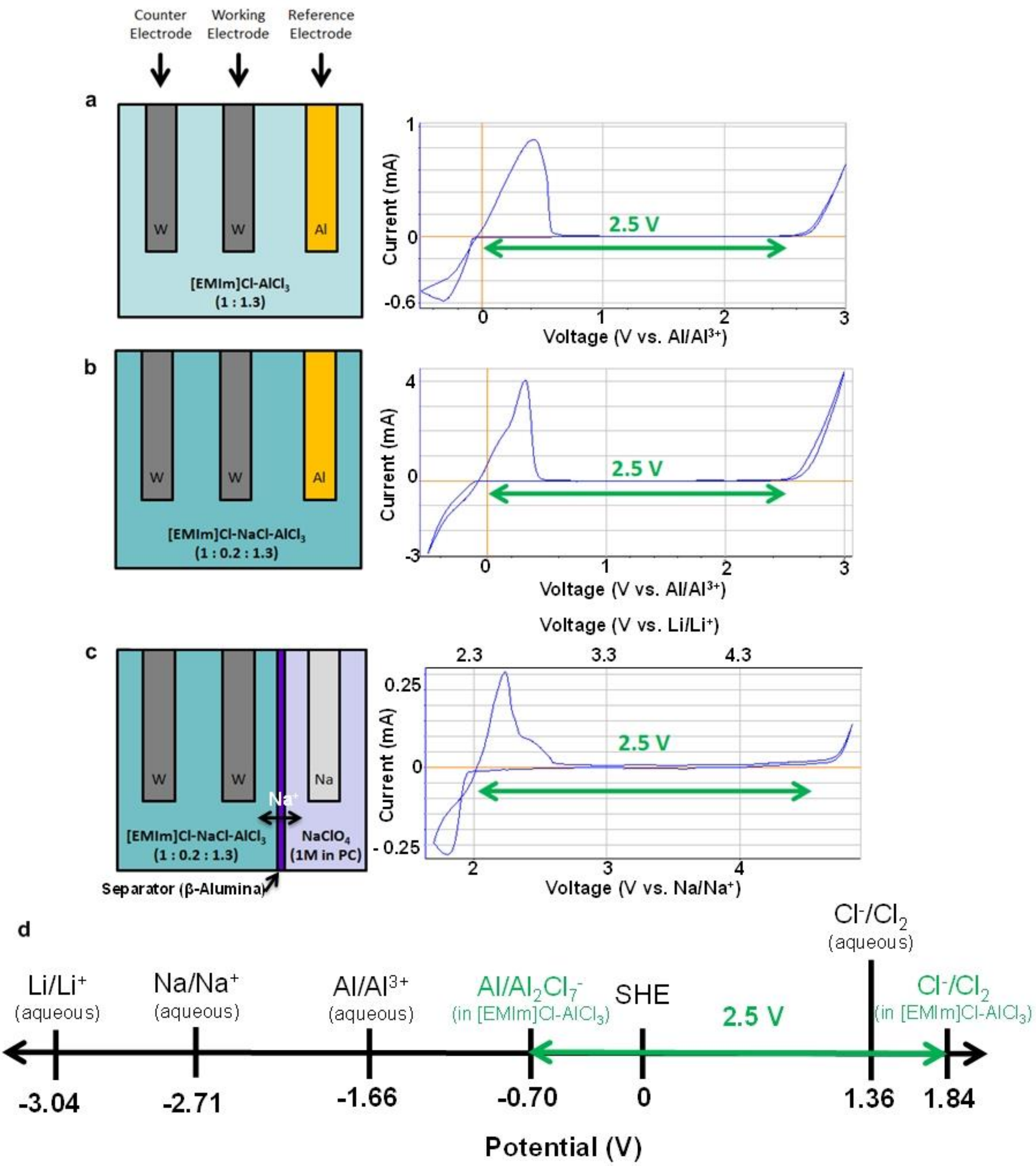

Figure S1. Three-electrode cyclic voltammetry measurements of aluminum stripping/ electroplating in (a) $AlCl_3$:EMIMCl (1:1.3 mol. ratio) ionic liquid; (b) $AlCl_3$:NaCl:EMIMCl (1:0.2:1.3 mol. ratio) ionic liquid; (c) $AlCl_3$:NaCl:EMIMCl (1:0.2:1.3 mol. ratio) ionic liquid and Na electrolyte (1M $NaClO_4$ in PC). (d) Schematic of electrochemical window of $AlCl_3$:EMIMCl (1:1.3 mol. ratio) ionic liquid, referenced to standard hydrogen electrode. Aqueous standard electrode potentials of Li, Na, and Al are indicated for comparison, highlighting high electrode potentials involved into operation of the Al battery.

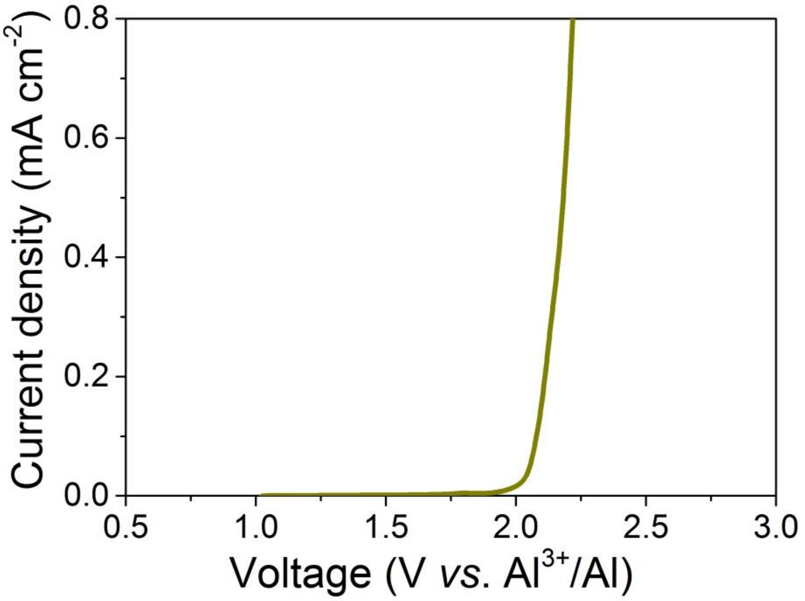

Figure S2. Cyclic voltammetry curve of Au current collector measured in $AlCl_3$:EMIMCl ionic liquid (r = 2) at a speed of 10 mV s^{-1}.

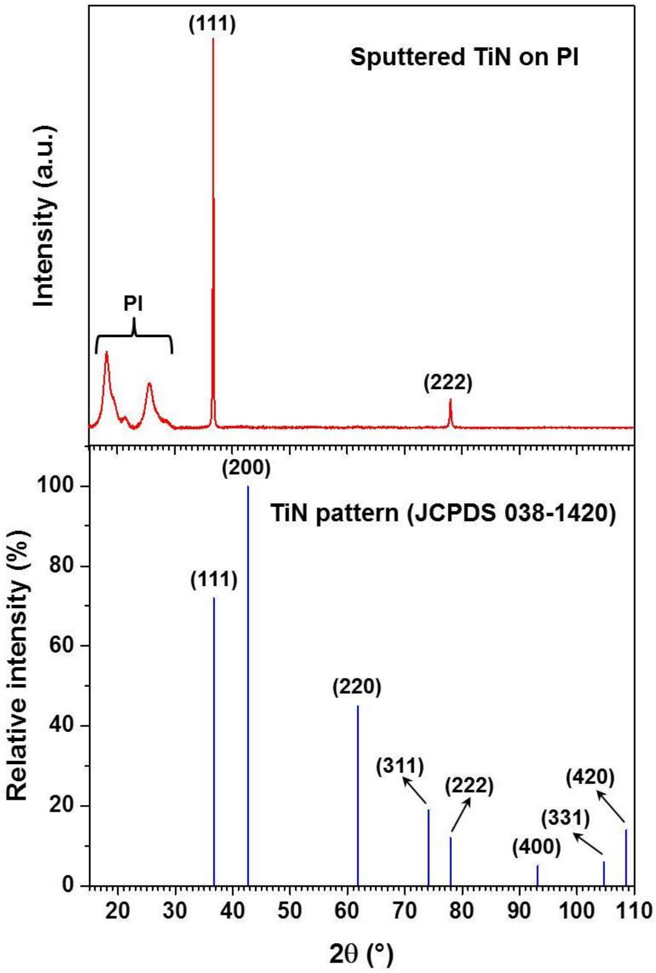

Figure S3. XRD patterns of TiN (1 μm thick) deposited on polyimide substrate (PI).

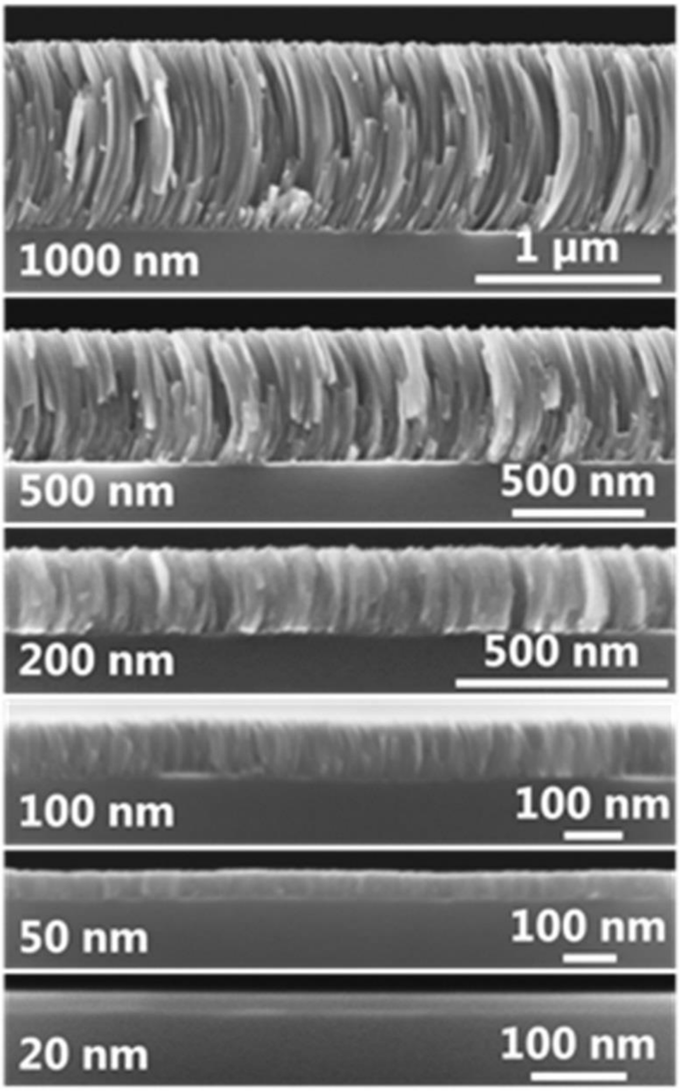

Figure S4. SEM cross section of TiN prepared by reactive magnetron sputtering on Si substrate.

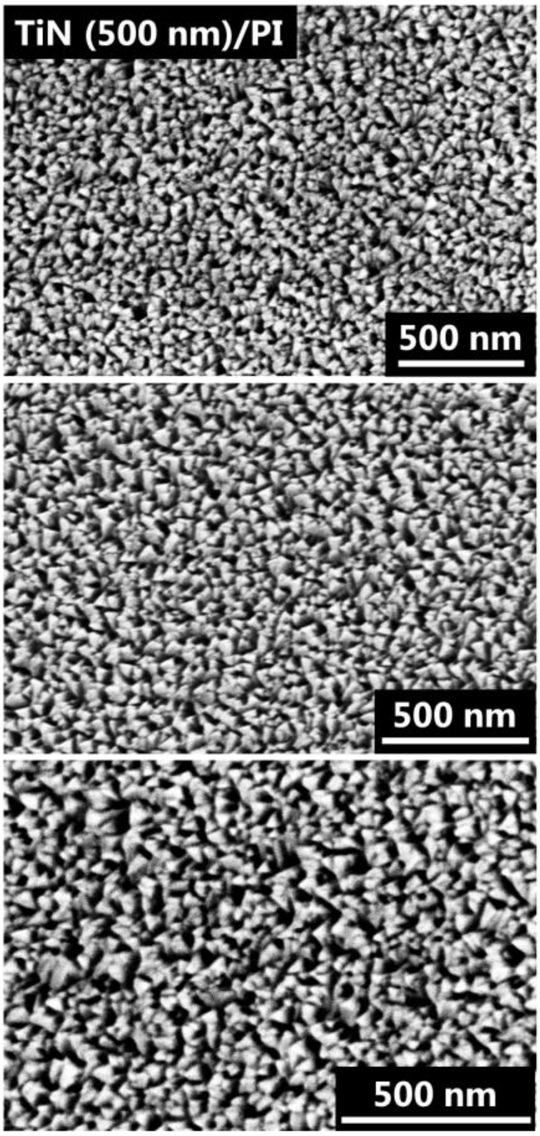

Figure S5. SEM images of TiN surface at different magnifications deposited onto polyimide substrate. Although the films are rough, they exhibit no pin holes or cracks.

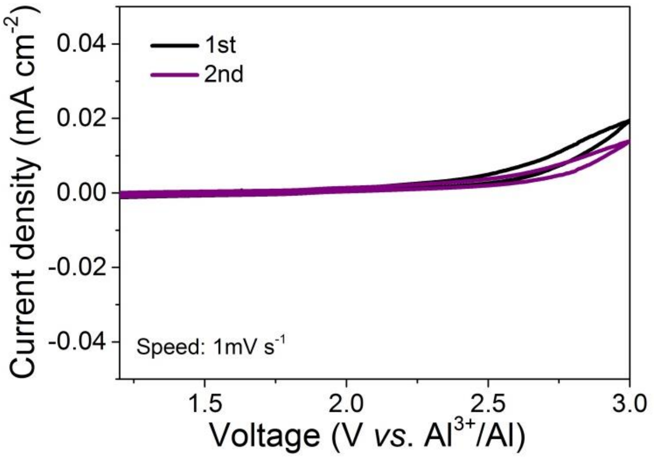

Figure S6. Cyclic voltammetry measurements of TiN current collector in $AlCl_3$-EMIMCl ionic liquid electrolyte (r = 2) using speed of 1 mV s^{-1}.

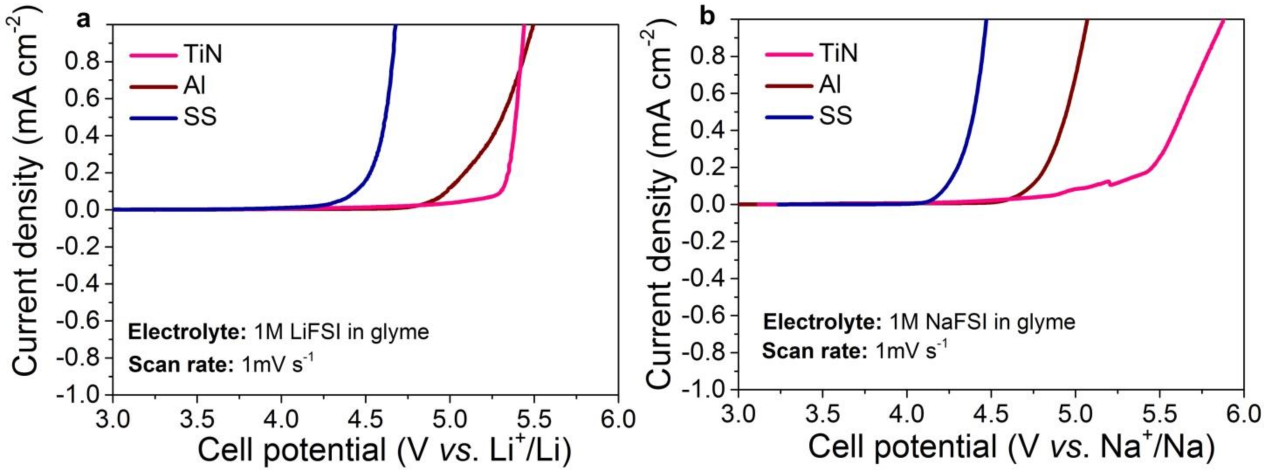

Figure S7. Cyclic voltammetry measurements of TiN, aluminum and stainless steel (316L) current collectors in (a) 1M LiFSI and (b) 1M NaFSI glyme based electrolytes.

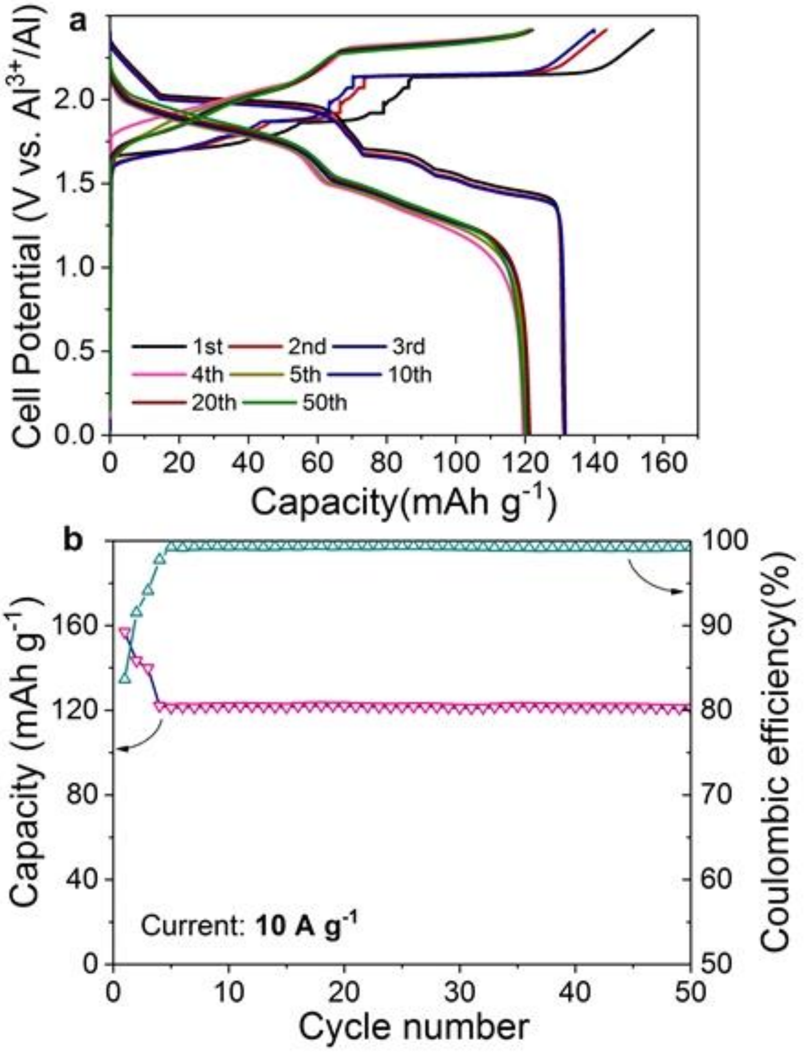

Figure S8. Electrochemical performance of kish graphite flakes in a pouch-type cell with a TiN current collector. (a) Galvanostatic charge-discharge voltage curves measured by a CCCV protocol at 50 mA g^{-1} (for first three cycles) and at 10 A g^{-1} (for the following cycles) in $AlCl_3$-[EMIM]Cl (r = 2). (b) Cyclability of kish graphite flakes measured by a CCCV protocol at 10 A g^{-1} (first three cycles were measured at 50 mA g^{-1}).

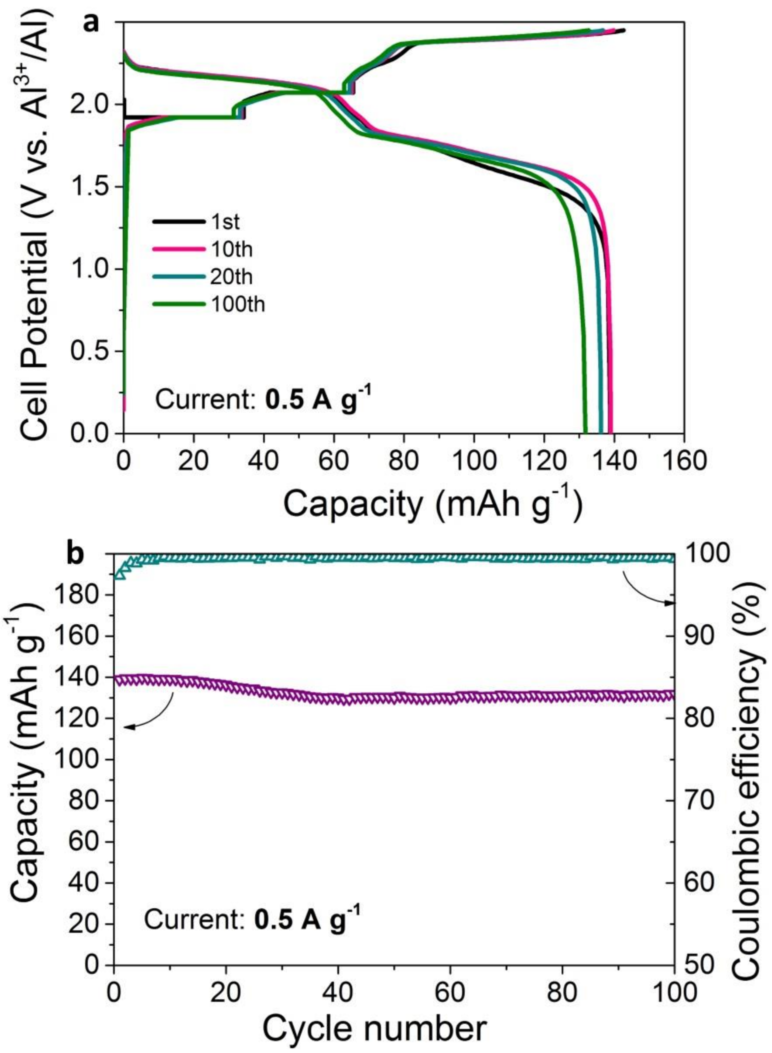

Figure S9. Electrochemical performance of kish graphite flakes in coin-type cell configuration with Cr_2N current collector. (a, b) Galvanostatic charge–discharge voltage curves and cyclic stability of kish graphite flakes measured with CCCV protocol at 0.5 A g^{-1} using using $AlCl_3$/EMIMCl ionic liquid (r = 1.3).

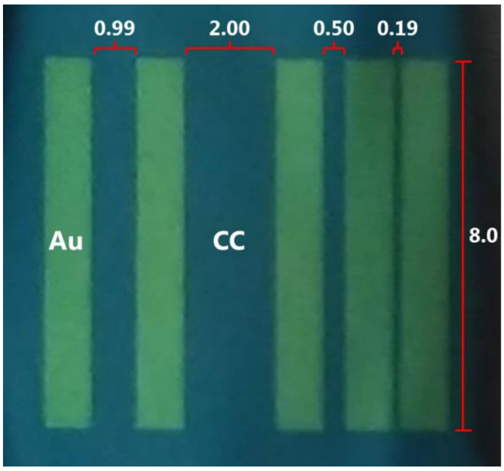

Figure S10. Photograph of 200 nm gold contacts deposited onto TiN (500nm thick, distances are expressed in mm).

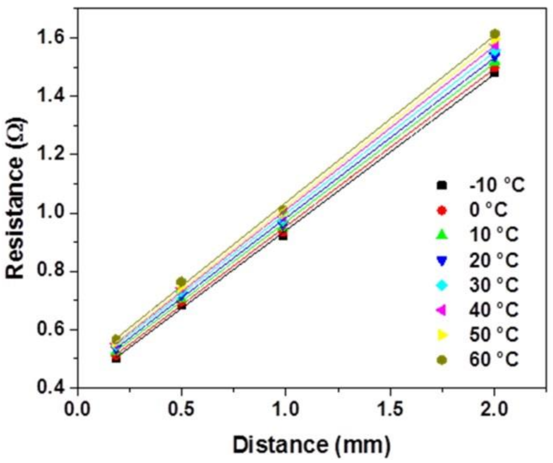

Figure S11. Electrical resistance vs. distance for TiN (500nm thick) in the temperature range –10 °C to 60 °C.
